# Determinants of hospital death in haematological cancers: findings from a qualitative study

**DOI:** 10.1136/bmjspcare-2016-001289

**Published:** 2017-06-29

**Authors:** Dorothy McCaughan, Eve Roman, Alexandra G Smith, Anne Garry, Miriam Johnson, Russell Patmore, Martin Howard, Debra A Howell

**Affiliations:** 1 Epidemiology & Cancer Statistics Group, Seebohm Rowntree Building, University of York, York, UK; 2 Department of Palliative Care, York Hospital, York, UK; 3 Centre for Health and Population Sciences, University of Hull, Hull, UK; 4 Queens Centre for Oncology and Haematology, Castle Hill Hospital, Cottingham, UK; 5 Department of Haematology, York Hospital, York, UK

**Keywords:** Haematological malignancies, leukaemia, lymphoma, myeloma, hospital death, end of life.

## Abstract

**Objectives:**

Current UK health policy promotes enabling people to die in a place they choose, which for most is home. Despite this, patients with haematological malignancies (leukaemias, lymphomas and myeloma) are more likely to die in hospital than those with other cancers, and this is often considered a reflection of poor quality end-of-life care. This study aimed to explore the experiences of clinicians and relatives to determine why hospital deaths predominate in these diseases.

**Methods:**

The study was set within the Haematological Malignancy Research Network (HMRN—www.hmrn.org), an ongoing population-based cohort that provides infrastructure for evidence-based research. Qualitative interviews were conducted with clinical staff in haematology, palliative care and general practice (n=45) and relatives of deceased HMRN patients (n=10). Data were analysed for thematic content and coding and classification was inductive. Interpretation involved seeking meaning, salience and connections within the data.

**Results:**

Five themes were identified relating to: the characteristics and trajectory of haematological cancers, a mismatch between the expectations and reality of home death, preference for hospital death, barriers to home/hospice death and suggested changes to practice to support non-hospital death, when preferred.

**Conclusions:**

Hospital deaths were largely determined by the characteristics of haematological malignancies, which included uncertain trajectories, indistinct transitions and difficulties predicting prognosis and identifying if or when to withdraw treatment. Advance planning (where possible) and better communication between primary and secondary care may facilitate non-hospital death.

## Introduction

The UK’s National End of Life Care Programme aimed to ensure individuals have more choice about where they die.^1^ The hierarchy of preferences most commonly reported rank home death first, hospice second and hospital the least favoured.^2^ While achieving home death is considered a key performance indicator and a proxy for quality end-of-life care, death in hospital is generally perceived as overly aggressive and suboptimal, along with emergency presentations, hospital admissions, chemotherapy treatments and intensive care input in the last weeks/days of life.^3 4^

Studies examining such issues report that patients with haematological malignancies (leukaemias, lymphomas and myeloma) are more likely to receive aggressive end-of-life care than those with other cancers.^5–8^ This includes an increased propensity for hospital death, a situation identified across countries, regardless of healthcare systems, disease subtypes and patient characteristics,^9 10^ reduced chance of palliative care and hospice referral or referral very close to death^11–14^ and (in some countries) particularly traumatic end-of-life experiences.^15^

Haematological malignancies are the fifth most common cancer in the UK.^16^ These are complex diseases, and although they have some similarities with other cancers, they often require different management strategies. Surgical resection is not an option, for example, and while some subtypes are curable with intensive, toxic chemotherapy and long periods of hospitalisation, others are incurable from diagnosis but managed with intermittent or continuous chemotherapy.^17^ Taking such factors into account, we explored the experiences of clinical staff and relatives of patients who had died from these cancers, to determine reasons why hospital deaths predominate.

## Methods

The study is set within the UK’s Haematological Malignancy Research Network (www.hmrn.org),^16^ an ongoing programme of work providing infrastructure for evidence-based research and aiming to improve the experiences of patients and their families. We conducted a qualitative study to elicit participants’ views, as this approach is well suited to exploring poorly understood phenomena.^18^

In-depth, semistructured interviews were carried out between 2012 and 2014 with 45 clinicians involved in the delivery of end-of-life care to patients with haematological malignancies, including nine haematologists, eight haematology nurses, six palliative care/hospice doctors, seven community-based and seven hospital-based palliative care nurses and eight general practitioners (GPs). Ten relatives of deceased patients were also interviewed; four of these patients had leukaemia, four had lymphoma and two had myeloma. They were all aged between 66 and 84 years at death and four were female. Recruitment involved purposive sampling,^18^ which ensured inclusion of clinicians from primary and secondary care, as well as relatives of people with a range of diseases. Potential interviewees were approached via email or by post.

Interviews lasted 30–90 min, were conducted privately in a hospital or university setting, or in relatives’ homes, and were audiotaped and transcribed. A topic guide (see online supplementary appendix 1) was developed from existing literature and experiences of the study team, but was used flexibly to allow for unanticipated responses. Data collection continued until no new information was forthcoming.^19^ Transcripts were analysed for thematic content. Coding and classification of data was inductive, following the sequential steps of data familiarisation by reading/re-reading transcripts, development of a coding frame to apply to the whole data set, attribution of data to individual codes, collating codes into themes and interpretation through seeking meaning, salience and connections.^20^ To promote transparency, rigour and trustworthiness,^20^ an independent qualitative researcher checked and corroborated the coding of five interviews (around 10%).

10.1136/bmjspcare-2016-001289.supp1 Supplementary Appendix 1

## Results

Based on their experiences, none of the clinical participants were surprised that patients with haematological malignancies were more likely to die in hospitals than people with other cancers. Five themes were identified: four relating to reasons for hospital deaths and one describing suggested changes to practice to facilitate non-hospital death when preferred. These themes are illustrated below using verbatim quotes.

### Theme 1: characteristics and trajectory of haematological malignancies

It was generally agreed that hospital deaths were principally determined by the characteristics and trajectories of haematological malignancies. These were considered complex and uncertain, with indistinct transitions between curative/life-prolonging and palliative approaches to care, leading to difficulties determining the appropriate time to initiate discussions about end-of-life care. This uncertainty was captured in descriptions of trajectories, which were said to range from gradual decline (often punctuated by episodes of acute deterioration) to sudden and unexpected death. The propensity for the latter was recognised as greater in patients with diseases requiring urgent, intensive treatment (eg, acute leukaemia or aggressive lymphoma) but was also said to occur on a background of gradual decline, typically in indolent diseases following remitting/relapsing pathways (eg, myeloma, chronic lymphocytic leukaemia and follicular lymphoma). Even within these categorisations, however, variations were found. Transitions were considered less dichotomous than in other cancers, where patients were described as being more likely to be discharged from individual specialisms at specific pathway points, typically after surgery or failed chemotherapy.

‘it could be severe neutropenic sepsis, rapid deterioration in a situation where you are trying to treat (the cancer), where somebody goes from well, to critically unwell, to dead, and you can’t plan for that, they suffer some sort of complication related to chemotherapy, so bleeding, internal haemorrhage, because they are profoundly thrombocytopenic…you can’t plan ahead for those (events)….’ (Haematologist 6)

‘some patients will deteriorate over days, some will last several months, even with acute leukaemia…’ (Haematologist 4)

‘I do think their disease trajectory makes it more difficult to, eh, actually predict when they’re going to sort of start deteriorating, compared to other cancers’ (GP 6)

Difficulties determining prognosis were discussed by many interviewees. This was complicated by pathways that might include sudden deaths or ‘dip up and down’ as multiple relapses and recoveries occurred. While the need for realistic, frank and early discussions of prognosis and the potential for sudden changes were considered crucial to advance planning, haematology staff described how these could sometimes only be initiated when patients had accepted that cure may not be possible. A further difficulty was identifying the point at which response to treatment (intensive/non-intensive chemotherapy or supportive care) was no longer likely to be achieved, particularly when patients may have recovered previously, even from seemingly dire situations.

‘the trouble is not knowing in advance what might be achieved and what might not…that’s quite tricky’ (Haematologist 9)

The frequent introduction of novel therapies for some diseases further complicated the issue of determining when to stop treatment. Treating patients aggressively in the last month and days of life was considered unavoidable, however, when this was being delivered with curative intent and sudden death occurred. In such situations, the transition to end-of-life care was described as absent, particularly among younger people, as emphasis was on saving life, often in intensive care settings. Treatment was also routinely given in the terminal phase as an intentional strategy for disease and symptom control, and to maintain quality of life for as long as possible. Non-haematology specialists considered this approach overly aggressive, however, believing that it occurred due to reluctance on the part of (some) haematologists to discuss withdrawing treatment and possible death, factors cited as impeding decision-making and advanced planning.

‘some consultants treat, treat, treat’ (Palliative nurse 5)

‘I think haematology want to keep patients going as long as possible and they don’t want to break that bad news’ (Palliative nurse 1)

Overtreating of certain groups of patients, such as those with acute myeloid leukaemia (AML), was acknowledged by some haematologists.

‘I personally think we treat far too many people far too aggressively, that the thrust of AML is completely wrong, if the patient is over 60 or 70 you are never going to cure them. Taking a more palliative, quality of life approach would probably be better’ (Haematologist 6)

Some respondents alluded to ‘co-dependency’ between haematologists and their patients, describing haematologists’ reluctance to ‘give up’ exploring treatment options. In this context, patients contributed to the impetus for ongoing treatment and hospitalisation, being desperate to ‘try anything’ in the hope of achieving cure or postponing death. Patients were said to associate this with (possibly false) hope for continuing life and discharge from haematology to home or hospice with imminent death.

‘it might be quite hard to… be impassive… you [haematologist] think, well, we’ve pulled them back from a similar situation before, maybe we can do it again…and that has some impact on your ability to let go…in the context of withdrawing treatment…up to that point, you might have always been the guy who had the next treatment ready, the next idea, if this doesn’t work, we’ll do this…and that has on occasion worked and helped improve quality of life or prognosis’ (Haematologist 4)

‘he was a fighting spirit…he wanted to go on being treated…he wanted treatment to the end’ (Haematology nurse 7)

‘she wanted anything, if it could prolong things, if it could give her more time, then she wanted to have it’ (Relative 9, concerning deceased wife)

‘we really wanted him to go to the hospice as he was dying, fading away and he would not have it…he chose to stay on the ward…he wanted to cling on to the blood tests…the reassurance he was still being watched’ (Haematologist 1)

### Theme 2: mismatch between the expectations and reality of home death

Patients’ and relatives’ limited knowledge and sometimes unrealistic expectations about end-of-life processes, and the sudden onset of distressing symptoms, such as bleeding and sepsis, were said to potentially precipitate a shift in preference away from home towards hospital death.

‘the reality of somebody dying at home can be very, very difficult and very different to the ‘home sweet home’ image you might have’ (Haematology nurse 4)

‘he would have continued at home if he hadn’t started coughing up blood and couldn’t get his breath’ (Relative 5—spouse died in the hospital)

As a consequence, another reported cause of hospital death was the frequency with which patients ‘bounce back’ into hospital due to sudden deterioration and distressing symptoms. Emergency calls from relatives, GPs or out-of-hours doctors were cited as major reasons for readmissions, as ambulance staff (often lacking information about preferences) were said to routinely transport patients to Accident and Emergency (A&E), resulting in hospital admission.

‘the problem is, if they’re bleeding and the relatives panicking, they call 999, obviously they end up going to A&E…then they end up with the problem of inappropriate treatments… they’re [emergency services] not aware of the fact that somebody’s got leukaemia in the terminal phase…we [primary care clinicians] need to make sure that we communicate well with A&E and the ambulance service, the paramedics, so they know exactly what they’re dealing with’ (GP 8)

### Theme 3: preference for hospital death

The close relationship between haematology staff, patients and their relatives was often discussed and was perceived to lead to preferences to remain in hospital at the time of death. This bond was considered a positive consequence of ongoing contact between the individuals involved over prolonged time periods. It was described as arising from experiences of ‘exemplary care’, in a setting often regarded as ‘a second home’, and associated with feelings of safety and security. Consequently, situations were described where remaining in the haematology ward, in a familiar environment with well-known staff, was said to be preferable to moving to a palliative care or hospice setting where new relationships would need to be built, often over short periods of time.

Some haematology staff shared similar sentiments, describing how transferring their patients to community staff they did not know for end-of-life care could feel like ‘abandoning’ them. These interviewees also described a certain satisfaction in being able to provide terminal care for patients they had known a long time, although this was sometimes countered by concerns about capacity (eg, staff time and availability of a private room).

‘you’ve been there for the relatives, for the carers and… you want to see all that through… the difficulty sometimes… is that by transferring somebody purely to palliative care, you almost feel as if you’re washing your hands of them’ (Haematology nurse 4)

‘I think that a lot of it… is building relationships again, and the way we work is that we, we see them at diagnosis and we do…all the chemotherapy treatments, so we get a really close bond and then they, they’re always coming back to clinic and we’re seeing them so if they relapsed, it would be us that they would see again ….so really in the end we’ve got kind of years of quite a strong bond with our patients and I think that’s the hard thing then is that when they do come towards the end I think they feel that they want to stay with us’ (Haematology nurse 7)

‘there’s only two people on nights for 20 patients, and if you’ve got someone who’s dying… it’s awful. During the day isn’t so bad… you have five nurses on, in the morning, and you go to two at night. So you know, it’s a big difference.’ (Haematology nurse 1)

Haematology doctors and nurses often questioned whether a good death could only occur at home, proposing instead that patients’ and their family’s needs for physical and emotional support could be effectively met through hospital haematology services, with input from palliative medicine as required, and that this may be entirely appropriate.

‘the current rhetoric that the only good death is a home death is absolute nonsense… it’s wherever the patient’s needs are best met… some patients get to know the nursing staff pretty well, and for them, it’s much nicer for them to go back to a ward where they know all the staff, than it is to go somewhere else or even to struggle in their home…so it may be their choice that they’re coming in to die, and I don’t think we should deny them that just because the Government has a bee in its bonnet about ‘people must die at home’… and I can see in haematology patients who have been coming in and out of a ward for years… a hospital death may be an appropriate death…and it may be done very well, actually’ (Haematologist 1)

Some differences were identified between haematology practitioner perceptions and those of other clinicians and some relatives, however, with the latter being most likely to cite home as the expected end-of-life preference.

### Theme 4: barriers and facilitators to home or hospice death

A number of practical barriers to non-hospital death that were specific to patients with haematological malignancies were identified. Some generalists expressed unease about managing haematology patients at home, either because they thought they themselves lacked knowledge about what were perceived as complex diseases, or because they considered that distressing symptoms, such as bleeding, were a barrier to death at home—a view shared by some haematology staff. Others, however, reported the relative infrequency with which such symptoms occurred, and did not consider the end-of-life care needs of haematology patients to differ much from that of people with other conditions.

‘bleeding would be the big one for patients with HM…you would have to have robust family and carers to cope with that’ (Palliative doctor 5)

‘the last patient that I tried to manage at home, she had a big, uh, GI bleed at the end of the life, so she came in, because of that, cause her husband could not cope… with that’ (Haematology nurse 5)

‘we’ve got dedicated district nurses, that, I would have every confidence, they could look after that…haematological malignancy may, may be more dramatic, but you can get just as ill with other malignancies…pain, bleeding, breathlessness, agitation, confusion, all those things you get with any cancer…and you get with the haematological malignancy patients. **Interviewer: So it’s not actually that different from looking after patients with other cancers.** Um, no, not in terms of providing end of life care’ (GP 3)

Due to the potential for rapid deterioration and death within hours or days, home-discharge (when preferred) could be required at short notice. While some hospitals had systems in place to organise this (eg, integrated discharge teams), others did not. In the latter case, the haematology nurses found such discharges could be time-consuming and frustrating, particularly if systems could not be put in place before the patient became too ill to be moved. Similarly, hospice beds were not always available at short notice, and inconsistencies in policies concerning the delivery of transfusions (often said to be required to maintain quality of life) represented barriers to referral.

‘when you are trying to get someone home, it takes up an incredible amount of time…phone the hospice, phone the district nurses, get stuff in place, like a bed, sometimes you don’t have time to do it…and the patient is deteriorating in front of your eyes…sometimes, a lot of the time, you have to stop and say, you are too unwell to go home now. I think that’s one of the reasons people don’t die at home’ (Haematology nurse 1)

‘the blocking point for us is that often there is no bed in the hospice, or they can’t deal with transfusion needs’ (Haematology consultant 5)

Inadequate communication across the primary/secondary care interface was regarded as a significant challenge to home death by GPs and community palliative care nurses. Haematological malignancies were described as cancers that were generally managed solely by haematologists in the hospital setting, with patients being encouraged to contact the haematology unit (rather than their GP) as the ‘first port of call’ for advice about their disease. Although GPs received correspondence about hospital appointments and admissions, this process was said to cause them to lose contact with their patients, and know little of their likely prognosis.

‘we can feel out of the loop….you don’t know what’s going on’ (GP 4)

In such cases, GPs were sometimes not aware that fast-track hospital discharge may be required to facilitate home death, and they typically felt that they were left with little time to develop relationships with the patient or their relatives before the patient’s death.

‘it’s usually two faxes, one for us (GP) and one for the district nurses, to say this patient is coming home…we’ve done this, we’ve done that, there’s no more active treatment, they will need…and there’s a list of stuff, and that’s sometimes the first we know of it…’ (GP 1)

Other important barriers to home death were reported that are likely to be common across other cancers, including gaps in community nursing services (particularly overnight), patients living alone and/or without lay carer’s support and the ability of lay carers to manage at home, particularly in circumstances involving young children, or lay carers who were themselves frail or unwell.

Analysis of data from relatives’ interviews revealed that the patients who had died at home (table 1), in line with their wishes, held strong preferences about place of care and death, which were in accord with relatives’ preferences.  They also had a good level of support from family members (who were said to have actively intervened to promote their wishes and ensure these were achieved), had symptoms that could be managed at home, considered nursing services’ support to be adequate and/or had the financial means to ‘buy in’ private carers to plug gaps, and had a GP who was closely involved in their care. Again, many of these factors are likely to be common across patients with other cancers.

**Table 1:** Patients’ (reported) and relatives’ (expressed) PPD, changes over time and actual place of death

Study ID	Patient’s PPD	Relative’s PPD	Patient’s change of preference	Relative’s change of preference	Actual place of death
1	Home	Hospice	He ‘couldn’t have cared’ (after sudden deterioration)	Hospital death was ‘peaceful’ and care was good	Hospital
2	Hospice	Home	Home	Unchanged	Home
3	Not stated	Not stated	Not stated	Not stated	Home
4	Not stated	Hospital	Not stated	Not stated	Hospital
5	Hospice	Home	Not stated	‘with hindsight, hospital was the right place’ (after sudden deterioration)	Hospital
6	Not stated	Home	Not stated	Unchanged	Home
7	Home	Home	Unchanged	Unchanged	Home
8	Home	Home	Unchanged	Unchanged	Home
9	Home	Home	Unchanged	Unchanged	Home
10	Home	Unsure	Not stated	Hospital was the ‘right place’	Hospital

PPD, preferred place of death.

### Theme 5: suggested changes to practice to support home or hospice death

Interviewees suggested a number of changes that they considered could facilitate home/hospice death. Early initiation of frank conversations about prognosis (where possible), alongside timely integration of haematology and palliative care specialists (including at diagnosis or while active treatment was still ongoing), was considered important by all interviewees. While some discussed difficulties identifying the appropriate time to involve palliative care clinicians, others considered mutual understanding of their roles as underpinning timely referrals. Cited methods of promoting joint working included co-location of facilities, shared clinics and integration of palliative care staff into haematology multidisciplinary team meetings. Interviewees described many such systems as already being in place, and most reported increasing collaboration over time.

‘we’re now in the same building…at the moment on the (palliative care) inpatient unit we’ve got two people with haematological malignancies here now and I’ve got one or two on the books…in the past my experience was that we got very few referred…now we’re all in one unit…we’re getting referrals through’ (Palliative doctor 5)

‘there is merit I think to look at scoping the need or benefits of having a joint clinic between palliative care and haematology to help identify those patients…who need palliative input’ (Palliative nurse 5)

‘over the time I’ve worked in this area, over 9 years, I think that it’s changing, haematologists are more readily involving palliative care at an earlier stage’ (Palliative nurse 3)

Another common suggestion was for consistent, clear and detailed documentation of end-of-life discussions, and electronic transfer of this across care interfaces. Primary care practitioners discussed the Gold Standards Framework^21^ (a system that many of the study hospital-based clinicians were unaware of) and how use of the surprise question (‘would you be surprised if this patient were to die in the next few months, weeks, days?’), may help to identify patients nearing end of life.^22^ It was largely agreed that such a system would facilitate prognostic estimations in secondary care, and if communicated to primary care, may enable fast-track discharge. It was considered limited, however, in the context of sudden death.

‘communication between the teams, secondary and primary care, and also of course from the haematology team as well, is essential…we need a clear indication—this is where we are and what we are or not doing, and this is what we might or might not be able to do with this patient—is a key, a key thing I think’ (Palliative doctor 4)

‘oncologists, when they’re writing to the GPs with clinic letters… there is now a drive for them to say, it would be appropriate for you to put this patient on your Gold Standards Framework register…um, that’s actually happening, I’ve seen it’ (Palliative doctor 5)

Other suggestions include early mobilisation of community-based services (district, Marie Curie and Macmillan nurses and ‘hospice at home’ services), and if preference is for home care and death, the setting up of mechanisms for anticipatory prescribing and arranging the installation of equipment (eg, hospital beds, syringe drivers).

## Discussion

Hospital deaths among patients with haematological malignancies were largely determined by the characteristics and pathways of these diseases, which resulted in uncertain trajectories, indistinct transitions and difficulties predicting prognosis and identifying if or when to withdraw treatment, factors which complicated advance planning. Delivery of care defined as aggressive in other studies,^5–8^ such as treatment close to the time of death, was often considered unavoidable and appropriate in the context of blood cancers, for both disease and symptom control. This suggests that the criteria currently used to assess end-of-life care may not be appropriate for haematological malignancies, a notion alluded to by others, but not always agreed on.^23–25^

Much literature has emerged in recent years, both promoting home death and questioning whether this is always preferable to hospital death.^26–28^ We found patient/relative choice to be commonly cited as the reason for hospital death and this explains to some extent the large proportions of deaths previously reported in this setting across disease subtypes, and not only among those who die suddenly.^10^ The desire among haematology doctors and nurses to provide terminal care for patients they have known and treated for many years is understandable, particularly among experienced staff who are skilled in managing end-of-life issues, and in situations where specialist palliative care support is also available.

The close relationship between haematologists and their patients undoubtedly underpins the concepts of ‘co-dependency’ and ‘the co-production of optimism’, which stem from the emotional and relational ties that develop within medical contexts.^29 30^ This is particularly important in blood cancers, where the most common barriers to quality end-of-life care have been described as unrealistic patient and clinician expectations, and clinician concerns about taking away hope.^25^ Complex situations have been described where haematologists must attempt to maintain hope while supporting patients to aim for cure, yet concurrently prepare for death.^31^

Identifying the optimum time to discuss prognosis and end-of-life preferences with haematology patients and their families is crucial, yet often difficult to achieve due to the propensity for sudden deterioration and unexpected death. More accurate prognostic data, frank early discussions (possibly near to the time of diagnosis) about potential outcomes (good and bad) and the appropriateness of further treatment may generate more realistic expectations. Such discussions are, however, likely to be difficult for some people, regardless of timing and the communication skills of the clinician; and may be impossible if patients die unexpectedly.

Although haematologists have been criticised in the past for lacking integration with palliative care services,^17^ we were informed of significant and increasing co-working between these specialities, often from diagnosis. Death in the hospice setting posed particular difficulties, however, largely due to a lack of available beds when needed and inconsistencies in service provision (ie, availability of blood product transfusions and antibiotics).

Fast-track hospital discharges were particularly challenging for primary care practitioners to manage. Our findings highlight the importance of sharing information across interfaces as a prerequisite for informed decision-making by GPs, nurses, out-of-hours doctors and paramedics and the prevention of unwanted hospital (re)admissions. Communication of broad life expectancy estimates (eg, identification of patients who might be expected to die within 6 to 12 months) might also enable primary care practitioners to facilitate discussions about end-of-life choices, and prepare for the possibility of home death, should this be preferred. Decision-making could also be supported by the widespread roll out across care settings of electronic systems that facilitate the recording of preferences. Electronic Palliative Care Coordinating systems^32^ have been introduced since our data collection took place, which may address this need, although views on their utility are mixed.^33^

### Strengths and weaknesses

As far as we are aware, this is the first UK study to explore determinants of hospital deaths in patients with blood cancers. Qualitative data were gathered from a range of key informants (healthcare practitioners and relatives of deceased patients) and a broad range of views and experiences was obtained, facilitating cross-comparison of perspectives between groups of participants and the identification of challenges across healthcare settings. We acknowledge that including bereaved relatives may provide a limited understanding of patient preferences, and that non-inclusion of relatives from minority ethnicity groups, whose perspectives may differ due to cultural factors, could limit generalisability. Additionally, our study drew on information provided by the relatives of patients aged 66–84; the experiences of relatives of younger or older patients may differ. Due to the relatively under-researched nature of the topic, however, these issues may be considered less salient than that of ‘sensitising’ readers to novel information.^20^

### Comparison to other studies

Although we did not identify any other studies specifically examining determinants of hospital deaths across such a wide range of interviewees, our findings are similar to those of other UK and non-UK studies examining end-of-life care more generally.^23 34 35^ These studies also highlighted differences between haematological malignancies and other cancers, often caused by disease factors (eg, complex transitions and difficulties estimating prognosis), and resulting in late end-of-life discussions and hospital death. They also note the close relationships that develop between haematologists and their patients, and the variable impact this may have on discussing and determining approaches to care at the end of life, including reluctance to dispel hope and feelings of abandonment if patients are handed over to palliative care services. While other studies also reported patient preferences to remain in the hospital to die,^36^ the notion that it was possible to provide terminal care for haematology patients at home was also supported,^37 38^ including the possibility of successfully managing issues such as bleeding in the home setting.

Recent studies from the USA report some haematology practitioners viewing palliative care involvement with distrust, perceiving it to be antithetical to cancer care, concerned only with end-of-life care, and contraindicative to ongoing treatment; the name of the service was also considered a barrier to referral.^39 40^ With the exception of limitations to the supportive treatment offered in hospice settings (eg, transfusions), we mainly found evidence of increasing collaboration and co-working. This is supported in a UK study that identified ‘universally favourable’ reports among haematologists about palliative care services.^35^ Possible reasons for such variations include differing perceptions and practices between countries.

## Conclusion

Hospital deaths were largely determined by the characteristics of haematological malignancies, which included uncertain trajectories and indistinct transitions, and difficulties predicting prognosis and identifying if or when to withdraw treatment. Although end-of-life care for patients with these diseases is often considered aggressive compared with other cancers, the reasons for this may be appropriate and unavoidable. Nonetheless, there is scope to improve practice, including: better information about prognosis (where possible), earlier, frank discussions about likely outcomes and improved communication between care settings.
