# Supplementary material for: Determinants of hospital death in haematological cancers: findings from a qualitative study
Source: BMJ Support Palliat Care. 2017 Jun 29;8(1):78–86. doi: 10.1136/bmjspcare-2016-001289 (PMC5867428; doi:10.1136/bmjspcare-2016-001289)
Supplement: Supplementary Appendix 1 [file bmjspcare-2016-001289supp001.pdf]

Appendix 1 Interview topic guide

**Clinical staff were asked about:**<br>• their perspectives on place of care and death in patients with haematological malignancies<br>• reasons why hospital deaths predominate<br>• factors preventing and promoting care and death at home<br>• factors associated with the primary/secondary care interface<br>• key factors affecting whether people achieve their preferred place of care and death<br>• issues specific to haematology compared to other conditions<br>• key changes that could facilitate death at home or closer to home
**Relatives were asked about:**<br>• their experiences of discussing treatment failure<br>• their experiences of discussing preferred place of care and death, and changes over time<br>• whether preferred place of care and death was/was not achieved, and reasons for this<br>• factors preventing/promoting death at home<br>• factors resulting in death in hospital<br>• their perceptions of differences between preferred place of care and death<br>• key changes that could facilitate care and death in the preferred place
